# “Living with a question mark”: psychosocial experience of Portuguese young adults at risk for hereditary amyloid transthyretin amyloidosis with polyneuropathy

**DOI:** 10.1007/s12687-024-00717-8

**Published:** 2024-07-08

**Authors:** José D. Pereira, Catarina Costa, Andreia Santos, Marina S. Lemos, Jorge Sequeiros, Milena Paneque, Álvaro Mendes

**Affiliations:** 1 CGPP - Centre for Predictive and Preventive Genetics, IBMC - Institute for Cell and Molecular Biology, University of Porto, Porto, Portugal; 2 i3S - Institute for Research and Innovation in Health, University of Porto, Porto, Portugal; 3 ICBAS - School of Medicine and Biomedical Sciences, University of Porto, Porto, Portugal; 4 FMUP - Faculty of Medicine, University of Porto, Porto, Portugal; 5 Associação de Solidariedade Social “O Tecto”, Vila do Conde, Porto, Portugal; 6 FPCEUP - Faculty of Psychology and Educational Sciences, University of Porto, Porto, Portugal; 7 CPUP - Center for Psychology, University of Porto, Porto, Portugal

**Keywords:** Young adult, Amyloidosis, hereditary, transthyretin-related, Genetic counseling, Portugal, Qualitative research

## Abstract

This study is the first to explore the psychosocial experience of young Portuguese adults at genetic risk for hereditary amyloid transthyretin amyloidosis with polyneuropathy (hATTR-PN). The work focuses on the developmental peculiarities of their experience with the disease. Sixteen semi-structured interviews were conducted with young adults coming for pre-symptomatic testing (PST) at a single genetics outpatient center in Portugal. The data were analyzed qualitatively. The main findings suggest that four themes mark the psychosocial experience of the young adults interviewed. The first refers to the development of psychological representations, namely beliefs, mental representations, and social perceptions about hATTR-PN. The second regards the experienced and anticipated psychosocial impacts, namely, suffering, anxiety, and relief related to the disease. The third is related to using strategies such as performing PST, strategies focused on emotional regulation and the meaning of hATTR-PN, and social strategies to deal with these impacts over time. Finally, the fourth aspect concerns the perceived and expected support for the participants’ needs provided by social contexts, that is, family and genetic counseling. In a period of life also marked by qualitatively different characteristics and developmental tasks from other life cycle stages (e.g., identity explorations, instability, and independent decision-making), experience with the disease can add psychosocial challenges to young adults at risk for hATTR-PN. Genetic counseling practices and health policies can be optimized to respond to the psychosocial needs of young adults. Future research should deepen the understanding of the psychosocial experience of individuals and families with late-onset hATTR-PN to improve the clinical response in this population.

## Introduction

Hereditary amyloid transthyretin amyloidosis with polyneuropathy (hATTR-PN) is a rare multisystem disease predominantly involving the peripheral nervous system (Adams et al. 2019). It is considered endemic in regions like Portugal (Schmidt et al. 2018). hATTR-PN is an autosomal dominant disease caused by the accumulation of amyloidogenic transthyretin in organs and tissues (Adams et al. 2019), which is mainly the result of the presence of the Val30Met variant (Parman et al. 2016) in the *TTR* gene (Adams et al. 2019). Despite traditionally considered an early-onset disease (< 50 years) in Portugal (Adams et al. 2021), Inês et al. (2018) reported a significant increase in the average age of onset and an increase in the representation of patients with late-onset hATTR-PN associated with this variant (≥ 50 years). In any case, to prevent the disease from progressing to death, patients diagnosed with hATTR-PN may benefit from the early use of disease-modifying therapies (Holmgren et al. 1993; Bulawa et al. 2012; Gorevic et al. 2021). On the other hand, genetic counseling is recommended for family members of people affected by hATTR-PN (Obici et al. 2016). Within this scope, a protocol for genetic counseling and psychosocial support in the context of pre-symptomatic testing (PST) was implemented in 1995 at the Centre for Predictive and Preventive Genetics (CGPP), a clinical unit of a research institute at the University of Porto, in Porto (Portugal). The protocol can be offered to people at 50% risk for hATTR-PN to predict their chances of developing the disease in the future and includes (a) a pre-test neurological examination, a psychosocial assessment, and at least two genetic counseling sessions, (b) a PST results dissemination session; and (c) a psychosocial follow-up at 3 weeks, 6 months and a year after the results (Sequeiros 1996). In a retrospective study collecting data from the clinical files of users who requested PST over the first 20 years of the CGPP (i.e., from 1996 to 2015), Paneque et al. (2019) reported that individuals at risk for hATTR-PN were the youngest group and those with the highest request rate of PST at the center (compared to Huntington’s disease, Machado-Joseph disease, and other late-onset neurological diseases groups). This can be explained by the fact that the disease affects mainly young adults in Portugal (Inês et al. 2018).

Young adulthood, defined by Arnett (2000, 2007) as the developmental period from the ages of 18 to 29, represents a unique moment in the life course in terms of the content, quality, and mediums of communication with family, friends, and romantic partners. Specifically, young adults are in a period of life marked by (a) identity explorations (which involve crucial decision-making processes related to social life, e.g., romantic relationships and career choices); (b) changes in romantic partners, jobs, educational directions, and living arrangements; (c) independent decision-making; (d) an ambiguous feeling related to their developmental definition between the periods of adolescence and adulthood; and (e) optimism about the future (Willoughby et al. 2021). While not exclusive to young adulthood, these characteristics peak in this period, which can be impacted by additional challenges related to the psychosocial experience of hATTR-PN.

Several studies (e.g., González-Moreno et al. 2021; Lopes et al. 2018; Magliano et al. 2021) have contributed to a better understanding of the psychosocial experience of hATTR-PN and its implications for the lives of members of families with the disease. Specifically, the family has been considered by study participants as the main source of knowledge and learning about hATTR-PN (Leite et al. 2016; Paneque et al. 2019). Therefore, it is inevitable that the psychological representations (e.g., beliefs, mental representations, and social perceptions) constructed about the disease are related to their family experience (Leite et al. 2017b; Mendes et al. 2017). Additionally, although some studies (e.g., Lêdo et al. 2013; Lopes et al.^2018^; Matos and Carvalho 2015) have reported healthy adaptations to PST results and balanced functioning in families with hATTR-PN, there is evidence (e.g., Lopes et al. 2018; Matos and Carvalho 2015) that the experience with the disease has a psychosocial impact. Various strategies have been described by members of families with this type of disease as they deal with these impacts (e.g., Leite et al. 2017a; Moos 1984). These include PST, regulating negative emotions associated with hATTR-PN, constructing meanings that make it possible to manage the situation, and seeking social support. Even so, successful versus dysfunctional coping and adaptation to hATTR-PN can be influenced by the way the family models (behaviors), encourages, informs, and supports its members (Oliveira et al. 2017a, b, 2021) and by the role of genetic counseling as a psychoeducational support context in helping families with the disease (Lopes et al.2018a; Rolland 2012; Rolland and Williams 2005).

Thus, despite the extensive literature on the psychosocial experience of members of families with hATTR-PN, few studies consider the specific developmental characteristics and tasks of young adulthood. In Portugal, on the one hand, young adults have been the most affected group by the disease, and on the other hand, individuals at risk for hATTR-PN were the youngest group and those with the highest request rate of PST at the CGPP from 1996 to 2015. Consequently, studying the psychosocial experience of young Portuguese adults at genetic risk for the disease contributes to a better understanding of the topic.

This work seeks to help improve genetic counseling practices and contribute to health policies that better attend to the psychosocial needs of this population. Therefore, this study aims to fill this research gap by exploring the psychosocial experience of young Portuguese adults at genetic risk for hATTR-PN, specifying the developmental peculiarities of their experience with the familial disease and the PST process. To the best of our knowledge, this is a topic has not yet been reported in this population.

## Method

### Study design

This research was based on a constructivist worldview and followed a qualitative approach by conducting interviews and qualitative data analyses suggested by Creswell and Creswell (2018) and Tesch (1990). The study design was selected to explore and understand the multiple meanings of the psychosocial experience of young adults at risk for hATTR-PN, providing results with methodological integrity. This research was approved by the Committee for Ethical and Responsible Conduct of Research of the Institute for Research and Innovation in Health (i3S).

### Participants

The participants were recruited among individuals undergoing PST for hATTR-PN at the CGPP. Convenience sampling was used to select the individuals aged 18 to 29 years old. First, the clinical secretary shared informational materials about the study and its objectives. Next, those interested in participating were approached in person or by telephone by J.D.P. to clarify any doubts about the research and to schedule the interviews.

The mean age of the participants was 21.25 years (*SD* = 3.02). All the participants were of European ethnic origin, single, living in the northwest region of Portugal, and undergoing the PST for hATTR-PN. Additional sociodemographic information about the participants is described in Table 1.

**Table 1:** Participants’ sociodemographic information

Sociodemographic characteristics	*n*	%
Sex		
Female	10	62.5
Male	6	37.5
Age (in years)		
18	3	18.75
19	3	18.75
20	1	6.25
21	3	18.75
22	1	6.25
23	2	12.5
24	1	6.25
25	1	6.25
29	1	6.25
Education		
Basic education	4	25
Secondary education	6	37.5
Higher education	6	37.5
Occupational status		
Active	15	93.75
Inactive	1	6.25
Sex of the parent diagnosed or at risk		
Female	8	50
Male	8	50
Status of the parent diagnosed or at risk		
Living without symptoms	4	25
Living with symptoms	10	62.5
Deceased	2	12.5
Number of participants’ siblings		
0	4	25
1 or more	12	75

### Procedure

This work is part of a broader research project that began in 2016 on the psychosocial experience of Portuguese families with hATTR-PN. In this study, 13 face-to-face interviews were conducted in a private room at the CGPP and three telephone interviews using the CGPP’s landline, respectively. The interviews continued until saturation of the data relevant to the topic under study was reached (Charmaz 2006). J.D.P. conducted the semi-structured individual interviews throughout the PST protocol and before each participant’s test result disclosure session. The interviewer first collected sociodemographic and other disease-related information. Then, open and closed questions about the psychosocial experience of being at risk for hATTR-PN were asked. These questions covered topics such as: (a) motivation to take the PST (e.g., feelings, expectations, the value of genetic information, as well as personal and family involvement with taking the test), (b) psychosocial impacts of hATTR-PN (e.g., perceptions of health and illness, and adaptation to family disease), (c) the experience of talking to family members and health professionals about test results or genetic risks more broadly, and (d) psychosocial needs (e.g., support). When other issues emerged as salient, the interviewer encouraged the participant to clarify and elaborate on his/her arguments. The interviews lasted 19 to 62 min, with an average duration of 41 min.

Informed consent was obtained from all the participants included in the study.

### Data analysis

All the interviews were audio-recorded with the consent of the participants and then transcribed verbatim into European Portuguese by J.D.P., C.C., and A.S. The transcripts were then analyzed by J.D.P. following the qualitative data analysis process suggested by Creswell and Creswell (2018) and specific coding procedures proposed by Tesch (1990). Specifically, after organizing and preparing the data for analysis, J.D.P. read all transcripts to get a general sense of the information. Then, four transcripts were read and re-read, and ideas in the margin on each page were jotted down. All emerging topics that emerged were listed in columns, compared, and grouped by similarity. Afterward, these topics were formed into new columns, arrayed as major, unique, and leftover topics. Subsequently, the topics were abbreviated as codes, and these codes were then written next to each appropriate text segment of all the transcripts to see if new topics emerged. Next, the most descriptive wording for the topics was found and they began to turn into categories. After that, the list of categories was shortened by grouping topics that were related to each other. After that, a final decision was made on the abbreviations for each category name and the codes were alphabetized. When J.D.P. finished coding, the data material belonging to each category was assembled, and a preliminary analysis was performed. Furthermore, the existing data were re-coded when necessary. Lastly, the coding process generated themes that were shaped into a general description and are represented using a narrative passage to convey the findings of the analysis.

All identifying information was removed from the transcripts by assigning identification codes to the participants. This action sought to ensure the confidentiality and anonymity of the data. The codes include the participant’s unique number (e.g., P1) to identify the source of the quotes.

The production of findings with methodological integrity was ensured by incorporating multiple validities and reliability procedures into this research following the recommendations of Creswell and Creswell (2018) and Gibbs (2007). Examples of multiple validities are the triangulation of different participants’ perspectives, the use of rich and dense descriptions to convey findings, the presentation of discrepant information with the general perspective of the participants, spending prolonged time in the field under study, and the participation of C.C. and A.S. as peer debriefers. Reliability procedures include verifying transcripts and holding regular meetings between J.D.P., M.P., and Á.M. to discuss procedures.

## Findings

**Fig. 1:**
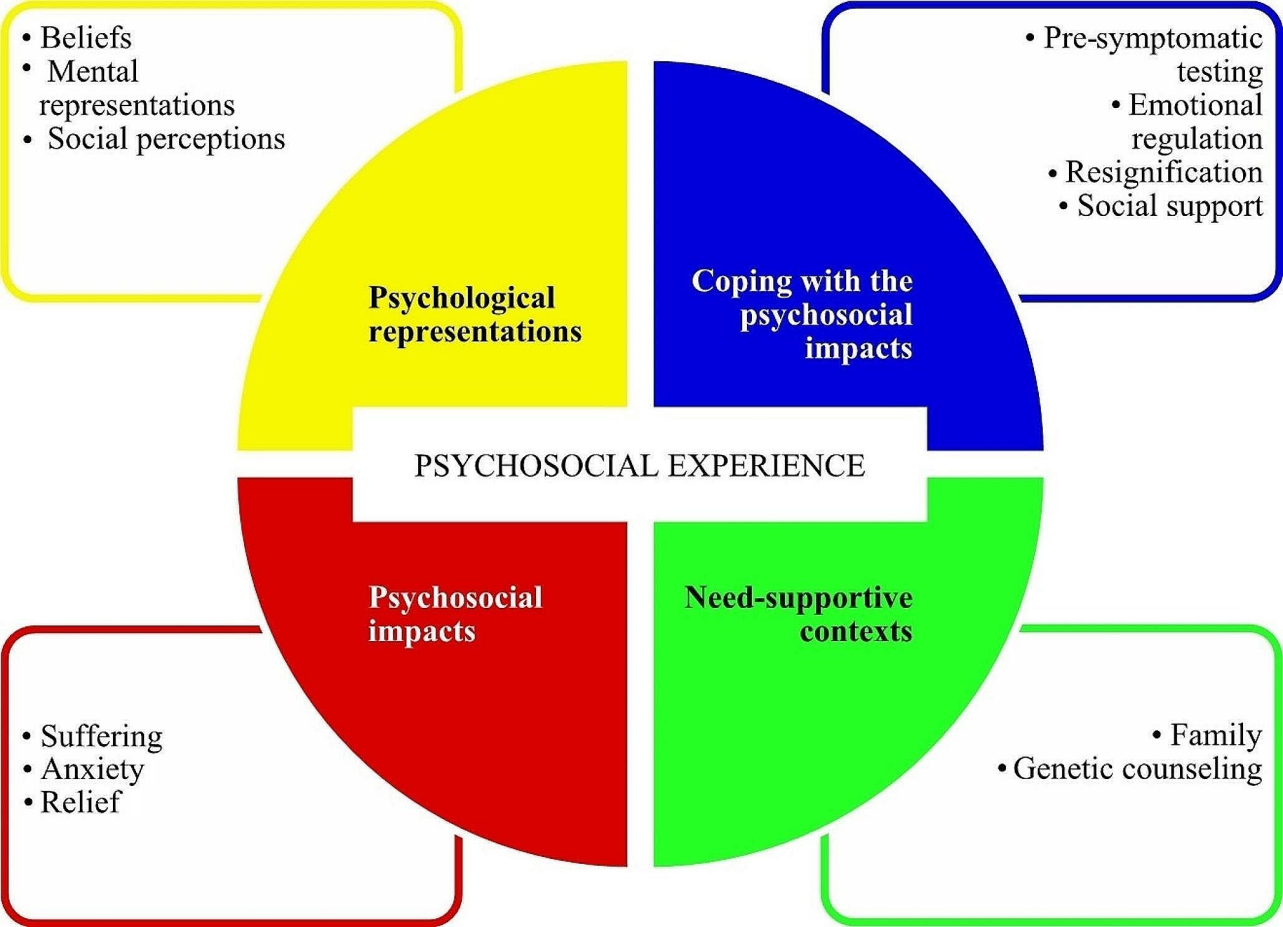
Themes (and their description) of the psychosocial experience of the young adults at risk for hATTR-PN interviewed

The data analysis suggested that the psychosocial experience of the young adults interviewed is marked by (a) the development of psychological representations about hATTR-PN, (b) experienced and anticipated psychosocial impacts related to the disease, (c) the use of strategies to deal with these impacts over time, and (d) the perceived and expected support for the participants’ needs provided by social contexts. These four themes, represented in Fig. 1, are not mutually exclusive and represent fluid categories.

### Psychological representations

The participants (e.g., P2) expressed having developed beliefs about the possibility of developing or not the disease and the factors that can influence this probability, as they experienced hATTR-PN on an individual and family level.

*P2: I think I’m going to get [the disease] because my brother has it too. I know that [the likelihood of developing it] is 50%, but it’s more likely that I’ll have it too.*

The participants’ psychosocial experience with hATTR-PN also led to the development of mental representations influenced by the different experiences of the disease and its consequences in the family context, as exemplified by excerpts from P11 and P5.

*P11: [hATTR-PN] isn’t something that scares me. I know it can have implications, but I think anyone who is informed and has seen all sides of the disease can think positively. If there is no cure, there are alternative responses that have been successful so far.*

*P5: [hATTR-PN] scares me a bit. I’ve never had much contact with the disease. My grandfather died of it, but I never met him. My mother has [hATTR-PN], but she’s 50 and has never had any symptoms. (…) I think [people with the disease] are normal. They just have a different gene.*

Participants (e.g., P1) also expressed having developed perceptions of social stigma related to hATTR-PN, especially in social contexts with little knowledge of the disease.

*P1: My family [thinks] like me, [they look at someone from a family with hATTR-PN as] a normal person. My family knows… Now, in society… sometimes even trying to distance themselves from the person because they have this disease is because they have no idea what [it] is. Because if they knew, maybe they would act in a normal way.*

### Psychosocial impacts

Participants (e.g., P12) said they had experienced episodes of suffering and anxiety as they experienced the disease and its consequences in a family context. Furthermore, they reported experiencing similar emotions associated with carrying out the PST and its possible results, as the excerpt from P9 illustrates.

*P12: [Being at genetic risk for hATTR-PN is to experience] the anguish of having or not having the disease and the pressure caused by the family to perform the PST. Sometimes [it’s] not being seen in the best light by society. [It’s experiencing] the suffering of seeing a father with the disease and everything he’s been through [because of it]. I think the accompaniment of all this kills us.*

*P9: I feel anxious [about taking the PST]. (…) I’m worried about my mother’s [reaction if the PST result is that I’m a carrier] and both my brother’s reactions [to the PST result]. It will be more complicated for me to convey to him that I don’t have the disease. But I think it will be more difficult for him to digest [knowing] that I have it. It’s a feeling… it’s going to be ambiguous.*

A possible non-carrier result in PST was anticipated by participants (e.g., P15) as an event that would generate individual and family relief, although a carrier result could produce similar emotions at an individual level due to the reduction in uncertainty associated with the genetic risk for hATTR-PN (as mentioned by P8).

*P15: [If the PST result is that I’m not a carrier] and allied to the fact that my brother doesn’t have the disease, that breaks a cycle. A cycle that is important to me but also brings peace to the family.*

*P8: [Whatever the PST result] I’ll be relieved. I won’t think about the 50/50 [chance of having or not having hATTR-PN] anymore.*

However, some participants (e.g., P5) said they had experienced reduced psychosocial impacts while living with the disease in a family context and reported that they expected similar impacts from the PST.

*P5: When I found out I might have [hATTR-PN], I didn’t know about it. I tried to get [the PST], but I was never worried about it. (…) My mother never had any symptoms, and if the disease appears, it will be late. (…) I know there are treatments [for hATTR-PN], so I don’t think there’s any reason to worry about that.*

### Coping with the psychosocial impacts

The participants (e.g., P9) expressed using the PST to cope with experienced or anticipated psychosocial impacts associated with hATTR-PN.

*P9: I’ve had enough of living with a question mark behind me. [Taking the PST] is to be sure [whether I’m a carrier of hATTR-PN or not] and to find out about my life. (…) [If the result of the PST is that I’m a carrier] it will give me time to get my head around it and to be more aware and more alert when I have symptoms [of the disease].*

The use of emotional regulation strategies (e.g., avoidance/distraction, catastrophizing, wishful thinking, rumination, and acceptance/resignation/disinvestment) was also expressed by participants (e.g., P7 and P5) as a resource to mitigate the psychosocial impacts experienced or anticipated concerning hATTR-PN.

*P7: When I’m feeling low, I try to distract myself straight away. (…) I think more about the fact that I’m going to have [a carrier result] to try to manage the response [to the PST result] better. (…) If we don’t both have [a carrier result] [i.e., the participant and a friend who also came to do the PST], it’s going to be a party all the way home. I’m thinking about that too, of course.*

*P5: [When I found out I might have the disease], I was almost always thinking [about it]. But then I didn’t spend much time thinking [about hATTR-PN]. If things have to happen, they will. (…) [The disease] is not the end of the world. There are solutions, and there will probably be new ones in a few years. (…) I’m not attaching too much importance to carrying out the test.*

In view of the impacts experienced, the participants (e.g., P11) also stated that they had given new meanings to hATTR-PN during the genetic risk state.

*P11: [When I found out about the disease in the family, it was] a bit scary (…) [Currently, hATTR-PN and the PST] is not something that scares me. (…) Being informed [about the disease] allows me to have a positive outlook on things. (…) I kept adding information [to what I already had], and [hATTR-PN and the possibility of developing it] was something that became natural.*

Participants (such as P12 and P5) also stated that they used social strategies (e.g., seeking support from significant others or avoiding talking about the disease outside their family context) to deal with the psychosocial impacts experienced or anticipated to be associated with hATTR-PN.

*P12: My mother clarified my doubts whenever I asked. Sometimes, I wondered whether I might have the disease or not, what the future might be like, and my mother answered these questions. [She] helped me to face [hATTR-PN] in a positive way.*

*P5: [There are people who] always have that idea of “You’ve got that disease, you poor thing.” That’s why there’s so much of that… trying to hide it, trying to let a small circle of people know about the disease because otherwise there will be that prejudice, that stigma. (…) [With the PST, the challenge is] dealing with people, being afraid that [they] will find out or know [about the family disease].*

### Need-supportive contexts

The support perceived by the participants for their psychosocial needs was influenced by different communication dynamics in their family contexts, as exemplified by excerpts from P11 and P10.

*P11: In my family, there is a very open view [about hATTR-PN]. (…) I was never pressured [by them] to do [the PST], they simply thought it was better for me to do it. (…) I live [the disease] with my family, I talk about it with my family, we live it like family. So, it belongs to everyone.*

*P10: The part of the family that might be carriers view [hATTR-PN] with contempt. They prefer to ignore the subject. (…) There’s a dilemma here. (…) I already know my parents’ and sister’s opinion [about doing the PST]. I already knew that their opinion was that I shouldn’t do it. And my boyfriend also thinks that maybe there’s nothing to be gained from doing it, but he respects my decision.*

Participants (e.g., P11) also considered genetic counseling as a context of psychoeducational support for families affected by hATTR-PN, not only because it raises awareness of the disease but also because of its role in supporting autonomy in carrying out the PST.

*P11: This journey [made at the CGPP when the PST was carried out], both in terms of information and psychological support, is beneficial [for the decision to carry out the test]. (…) The fact that we talk about this makes us more aware of the influence that screening and being diagnosed as positive [i.e., carrier] or negative [i.e., non-carrier of hATTR-PN] have.*

## Discussion

This study is the first to report the psychosocial experience of young Portuguese adults at genetic risk for hATTR-PN and extend previous scientific evidence on the experience of members of families with the disease (e.g., González-Moreno et al. 2021; Lopes et al. 2018; Magliano et al. 2021). The main findings suggest that four themes mark the psychosocial experience of the young adults interviewed. The first refers to the development of psychological representations, namely beliefs, mental representations, and social perceptions about hATTR-PN. The second regards the experienced and anticipated psychosocial impacts, namely, suffering, anxiety, and relief related to the disease. The third is related to using strategies such as performing PST, strategies focused on emotional regulation and the meaning of hATTR-PN, and social strategies to deal with these impacts over time. Finally, the fourth aspect concerns the perceived and expected support for the participants’ needs provided by social contexts, that is, family and genetic counseling.

For the young adults, it was mainly during their family experience with the disease that psychological representations of hATTR-PN were developed, as already reported in other studies (e.g., Leite et al. 2016; c; Mendes et al. 2017). Specifically, participants expressed having known the characteristics of the disease and its consequences through family experience with relatives affected by hATTR-PN, as previously described by Leite et al. (2017a, b, c) and Lopes et al. (2018). Nevertheless, some young adults overestimated the perceived risk in relation to the actual 50% risk of having or not having any of the genetic variants associated with the disease. Although, according to the results presented by Leite et al. (2017b), it may be mainly older adults who show an increased perception of risk compared to younger adults, it cannot be ruled out that young adults may also build beliefs about an increased individual probability of developing hATTR-PN. In fact, different experiences of family illness (e.g., how the disease developed or not in the family, or the existence or not of losses related to hATTR-PN) and the reflections they gave rise also to seem to have influenced the development of beliefs and mental representations on the part of the young adults interviewed. Despite this being a developmental period particularly focused on establishing young adults’ autonomy from their parents and exploring their identity (Willoughby et al. 2021), results highlight the family as the primary source of knowledge and learning about hATTR-PN (Leite et al. 2016; Paneque et al. 2019). It was also during their family experience with the disease that some participants said they had developed perceptions of social stigma associated with hATTR-PN, which can affect crucial choices typical of this period of life (e.g., romantic/reproductive decisions and career choices; Willoughby et al. 2021), a result previously reported by Mendes et al. (2017).

The experience of the family illness also had a psychosocial impact on the young adults interviewed. Specifically, monitoring the development and respective consequences (e.g., social stigma) of hATTR-PN in the family, as well as experiencing the process of undergoing PST, gave rise to episodes of suffering and anxiety in some participants, which is in line with what has already been reported in other studies (e.g., Lopes et al. 2018; Matos and Carvalho 2015; Mendes et al. 2017). Notably, in addition to the uncertainty about whether they have inherited the disease-causing genetic variant or not (which in it-self can impact the identity development typical of young adulthood; Willoughby et al. 2021), some participants reported feeling that their family exercised some control over their decision-making regarding PST, a result already described by Matos and Carvalho (2015). Older generations can play roles in promoting health and managing genetic risks concerning younger generations (Oliveira et al. 2017a, b, 2021), and this can be assumed to be a protective factor in families with hATTR-PN (Lopes et al. 2018). However, attempts by family members to control the individual choices of young adults can add psychosocial impacts to a developmental period generally marked by a growing autonomization of decision-making based on the interests and preferences of the younger individuals (Willoughby et al. 2021).

Furthermore, the emotional and relational impacts of the possible results of the PST, anticipated by young adults in this study, can influence changes in family dynamics and communication patterns (Lopes et al. 2018a; Matos and Carvalho 2015) and the development of perspectives on future life projects (Lopes et al. 2018a; Matos and Carvalho 2015) characteristic of this developmental stage. This possibility adds new challenges to an already unstable time of life (i.e., where there are often changes in love partners, jobs, educational directions, and living arrangements; Willoughby et al. 2021). Even so, as reported by some participants, a positive adaptation to a carrier or non-carrier result of hATTR-PN (Lêdo et al. 2013; Matos and Carvalho 2015), as well as a balanced functioning of families with the disease (Lopes et al. 2018) are possible. However, reduced impacts of the psychosocial experience with hATTR-PN, such as those already reported by Matos and Carvalho (2015), Rolim et al. (2006),) and others exemplified by a young adult in this study who is a member of a family with late-onset hATTR-PN (viz., P5), may pose additional challenges to clinical practice with this population (Inês et al. 2018). Specifically, individuals at risk for hATTR-PN may show a better response to PST than individuals who are at risk for other late-onset genetic neurodegenerative diseases because of the availability of disease-modifying therapies (Rolim et al. 2006). However, Lêdo et al. (2018) suggested that genetic counseling professionals should focus on psychologically supporting at-risk persons rather than paying more attention to the PST result.

As mentioned previously, the average age of onset and the representativeness of patients with a late-onset hATTR-PN have increased in Portugal (Inês et al. 2018). Additionally, a diminished multigenerational experience with the disease in individuals of families with a late-onset of the condition differs from the traditional life trajectories associated with hATTR-PN in the country (Lopes et al. 2018). These two facts may lead to a lack of information related to genetic risk and its associated psychosocial implications in members of families with late-onset hATTR-PN. According to Rolland and Williams (2005), this may provoke a reduced concern about the disease and, in effect, sharpest future emotional transitions as the individuals become aware of these genetic issues with a predictable impact on their developmental tasks.

Given the impacts experienced and anticipated, PST was the strategy favored by the participants to deal with the emotional and relational challenges associated with their genetic risk status for hATTR-PN, which is in line with what was reported by Leite et al. (2017a) and Matos and Carvalho (2015). More specifically, while undergoing PST can be a resource in the very exploration of the typical identity of young adults (Leite et al. 2017a; Matos and Carvalho 2015; Willoughby et al. 2021), a PST result can define the sense of mastery in coping with a treatable disease (Leite et al. 2017a; Matos and Carvalho 2015; Rolland 2012), which is supported by the accounts of some participants. In line with what was reported by Leite et al. (2016) and Leiteet al. (2017a, b, c), the psychosocial experience of participants in this study is also characterized by the use of other strategies, such as those focused on emotion, meaning-making and seeking social support (viz., in the three vital interaction systems of young adults: parents, friends, and romantic partners; Willoughby et al. 2021). For successful long-term adaptation to the disease, individuals and families benefit from regulating negative emotions associated with hATTR-PN (viz., gradually accepting the disease), creating meanings that support the feeling of mastery and competence, as well as seeking emotional or instrumental support from others (Moos 1984; Rolland 2012; Rolland and Williams 2005). Even so, following Rolland (2012) and Rolland and Williams (2005), the quality of the fit between the psychosocial challenges associated with the condition and the functioning and resources of the support contexts determines the quality of coping and adaptation to the disease.

Moreover, the perceived support for the participants’ needs provided by social contexts influenced their own psychosocial experience with hATTR-PN. Specifically, different family communication dynamics seemed to influence the support perceived by some participants to respond to a disease understood as familial and intergenerational (Rolland 2012; Rolland and Williams 2005). Bearing in mind that the family plays a vital support role in the various stages of adaptation to genetic risk and the disease, the experience with hATTR-PN can be influenced by how the family context (a) transmits the experiences of confronting and managing the disease; (b) facilitates or hinders the passage of and access to information about hATTR-PN; (c) encourages or discourages the implementation of risk management measures, early detection, and treatment; and (d) provides emotional and instrumental support (Oliveira et al. 2017a, b, 2021). Following the results presented by Oliveira et al. (2017a, b, 2021), it is then possible that these family dynamics may, in turn, affect the way young adults become aware of the disease and cope with it (e.g., whether they carry out PST or not), influencing the very exploration of identity typical of this developmental period (Willoughby et al. 2021). Furthermore, in a phase marked by active consideration of PST and, according to Willoughby et al. (2021), by increasing autonomy in the decision-making process, genetic counseling, as a psychoeducational support context, can help adjust the young adults’s awareness of hATTR-PN. The psychoeducation provided throughout the PST process can provide a vital support context to mitigating possible maladaptive impacts associated with the test results. In this process, genetic counseling professionals play a crucial role in helping families (and, particularly, their young adults) by providing a psychosocial understanding of the disease in practical, emotional, and longitudinal terms (Lopes et al. 2018a; Rolland 2012; Rolland and Williams 2005). Specifically, genetic counseling professionals can help young adults at risk for hATTR-PN in communication processes, decision-making, and developing contingency plans associated with their developmental tasks (viz., those related to their love/reproductive and professional lives; Rolland and Williams 2005). The professionals may also help family members acquire a common understanding of the biopsychosocial aspects of the disease, facilitating family communication that is more focused on coping strategies and adaptation to genetic risk and hATTR-PN.

### Strengths and limitations

The results reported in this study contribute to filling gaps in the understanding of the psychosocial experience with hATTR-PN in a population with unique developmental characteristics (Willoughby et al. 2021). Nonetheless, they should be carefully read and interpreted due to the small number of participants that characterizes qualitative research such as this. Additionally, although various validity and reliability procedures have been implemented to ensure the methodological integrity of the data, subsequent studies may incorporate additional analytical processes (e.g., conducting follow-up interviews with study participants and providing them with an opportunity to comment on the results, as well as introducing intercoder agreement processes), further strengthening the validity and reliability of their findings. Since the purpose of this research is restricted to the specific description of the psychosocial experience of young Portuguese adults at genetic risk for hATTR-PN in a particular spatiotemporal context, the transferability of these results to other populations, diseases, and contexts is limited. Furthermore, the sample studied included only young adults who underwent PST at the CGPP (excluding individuals with severe cognitive impairment), so further research could verify the generalizability of these data to the population of young Portuguese adults at genetic risk for hATTR-PN in general and in other countries.

### Implications

This study has implications for future research, policies, and practices. Firstly, an in-depth study on the psychosocial experience of members of Portuguese families with late-onset hATTR-PN can help extend the evidence reported in this research, filling gaps and optimizing the health services that support the individuals of families with the condition in the context of genetic counseling. Secondly, strengthening the implementation of collaborative policies between local health services and patient associations can help to enhance their important work in providing psychosocial support to patients and their families. Thirdly, this study provides clues that can contribute to optimizing the practice of genetic counseling with young adults by considering the developmental tasks and specific psychosocial needs of this population in a biopsychosocial intervention process. For example, psychoeducational or support groups can be designed to meet the needs of young adults at risk for hATTR-PN in coping with the various forms and stages of the disease, empowering the individuals for the psychosocial challenges of the illness and preventing risks of maladaptation to PST results.

## Conclusion

This study reports the first description of the psychosocial experience of young Portuguese adults at genetic risk for hATTR-PN. Given that the young adult population has been reported as the one most affected by the disease in Portugal, this research can provide important data to optimize genetic counseling practices and health policies that respond to the specific needs of this population. Moreover, the context of an increase in the average age of onset and the representativeness of patients with a late-onset hATTR-PN in the country may pose additional complex challenges for families with this form of the disease (and the young adults who are part of them) as well as for health systems. This highlights the importance of deepening our understanding of the psychosocial experience of affected individuals and families to improve the clinical response.

## Data Availability

The participants in this study did not consent for their data to be shared publicly, so supporting data is unavailable.

## Declarations

### Data transparency

J.D.P., C.C., A.S., M.S.L., J.S., M.P., and Á.M. declare that all data support their published claims and comply with field standards.
